# Effects of a multilevel intervention of resistance training with or without beta-hydroxy-beta-methylbutyrate in medical ICU patients during entire hospitalisation: a four-arm multicentre randomised controlled trial

**DOI:** 10.1186/s13054-023-04698-x

**Published:** 2023-12-15

**Authors:** Ting-Ting Wu, Qiao-Ling Chen, Xiu-Xia Lin, Mei-Lian Xu, Xue-Xian Chen, Chen-Juan Luo, Yao-Ning Zhuang, Yue-Qing Wei, Jing-Bing Wu, Jing Xiong, Li-Li Chen, Hong Li

**Affiliations:** 1 Shengli Clinical College of Fujian Medical University, Fuzhou, China; 2 School of Nursing, Fujian Medical University, No.1 Xuefu North Road, Minhou County, Fuzhou, 35001 China; 3 Department of Nursing, Fujian Provincial Hospital, Fuzhou, China; 4 Surgical Intensive Care Unit, Fujian Provincial Hospital, Fuzhou, China; 5 Cardiac Intensive Care Unit, Fujian Provincial Hospital, Fuzhou, China; 6 Intensive Care Unit, Longyan City First Hospital, Longyan, China; 7 Intensive Care Unit, Ningde Normal University Affiliated Ningde City Hospital, Ningde, China; 8 Intensive Care Unit, Nanning City First Hospital, Nanping, China; 9 Respiratory and Intensive Care Unit, Putan College Affiliated Hospital, Putian, China; 10 Respiratory and Intensive Care Unit, Fujian Provincial Hospital, Fuzhou, China; 11 Internal Medicine Intensive Care Unit, Fujian Provincial Hospital, Fuzhou, China; 12 Department of Nursing, Second Affiliated Hospital of Fujian Medical University, Fujian, China

**Keywords:** Intensive care unit, Resistance training, Beta-hydroxy-beta-methylbutyrate, Multicentre, Randomised controlled trial

## Abstract

**Background:**

Intensive care unit-acquired weakness (ICU-AW) is a prevalent and severe issue among ICU patients. Resistance training and beta-hydroxy-beta-methylbutyrate (HMB) intervention have demonstrated the potential to enhance muscle function in patients with sarcopenia and in older adults. The purpose of this study was to determine whether resistance training and/or HMB administration would improve physical function, muscle strength, and quality of life in medical ICU patients.

**Methods:**

In this multicentre, four-arm, single-blind randomised control trial, a total of 112 adult patients with internal medical diagnoses admitted to the ICU were enrolled. These participants were then randomly assigned to one of four treatment groups: the resistance training group received protocol-based multilevel resistance exercise, the HMB group received 3 g/day of HMBCa, combination group and control groups received standard care, from the ICU to the general ward until discharge. The primary outcomes assessed at discharge included six-minute walking distance (6MWD) and short physical performance battery (SPPB). Secondary outcomes measured included muscle mass, MRC score, grip strength, and health reports quality of life at different time points. Data analysis was performed using a generalised linear mixed model, adhering to the principles of intention-to-treat analysis.

**Results:**

Resistance training and combination treatment groups exhibited significant increases in SPPB scores (3.848 and 2.832 points, respectively) compared to the control group and substantial improvements in 6WMD (99.768 and 88.577 m, respectively) (all with *P* < 0.01). However, no significant changes were observed in the HMB group. Muscle strength, as indicated by MRC and grip strength tests conducted at both ICU and hospital discharge, showed statistically significant improvements in the resistance training and combination groups (*P* < 0.05). Nevertheless, no significant differences were found between the treatment groups and usual care in terms of 60-day mortality, prevalence of ICU-AW, muscle mass, quality of life, or other functional aspects.

**Conclusions:**

Resistance training with or without beta-hydroxy-beta-methylbutyrate during the entire hospitalisation intervention improves physical function and muscle strength in medical ICU patients, but muscle mass, quality of life, and 60-day mortality were unaffected.

**Trial registration:**

ChiCTR2200057685 was registered on March 15th, 2022.

**Supplementary Information:**

The online version contains supplementary material available at 10.1186/s13054-023-04698-x.

## Background

Intensive care unit-acquired weakness (ICU-AW) is a frequent problem that induces skeletal muscle wasting while patients are suffering from a life-threatening condition [1]. The prevalence of ICU-AW was found to be 48% [2]; furthermore, muscle weakness persisted in 50% of these patients during the five-year follow-up period [3]. ICU-AW yields significant clinical implications that profoundly impact rehabilitation outcomes, with prolonged length of stay, delayed weaning from mechanical ventilation, extended morbidity and mortality rates, and impaired quality of life [1]. According to a research agenda [4] for this condition, the foremost research priority in preventing ICU-AW is to investigate the interaction between early rehabilitation and nutritional therapy.

Emerging research indicates the significant influence of beta-hydroxy-beta-methylbutyrate (HMB) on skeletal muscle mass and physical function in a clinical context, such as in the case of sarcopenia, hospitalised elderly patients, cancer cachexia, and critical illness [5]. This effect can be attributed to HMB's mechanism of action, which involves stimulating muscle protein synthesis [6, 7] and inhibiting protein degradation by blocking the ubiquitin pathway [8]. Nevertheless, the effectiveness of HMB application in critically ill patients exhibits noteworthy heterogeneity [9–13]. The limited efficacy of individual HMB nutritional intervention in ICU patients may result from their constrained physical activity levels and inadequate exercise-induced stimulation of muscle protein synthesis [13]. Therefore, further validation is required to establish the potential efficacy of a combined intervention involving HMB supplementation and exercise in achieving the intended outcomes.

RT is another non-pharmacological intervention that has been studied extensively in a variety of healthy [14] and clinical populations [15] to directly or indirectly support muscle protein turnover [16]. RT has been shown to activate skeletal muscle, augment myofibrillar protein accretion, suppress muscle breakdown, and possess anti-inflammatory properties in older adults [17, 18]. These research findings suggest benefits in mitigating muscle atrophy and enhancing muscle strength, which could also prove highly advantageous for critically ill patients. Previous studies have substantiated the feasibility and safety of incorporating low-intensity RT as a component of multimodal interventions in patients admitted to ICU [19, 20]. However, elucidating the distinct effects of RT alone on ICU patients remains challenging within the field of multi-component exercise training.

Therefore, the aim of the present study was to investigate the potential benefits of RT and/or HMB administration in internal medical critically ill patients. To achieve this goal, we conducted a multicentre, assessor-blinded, four-arm randomised controlled trial (RCT) comparing RT alone, HMB alone, the combination of both interventions, and standard care throughout the entire hospitalisation intervention period, including ICU and general ward stays. The hypothesis of this innovative RCT posits that the combined intervention leads to (1) enhanced physical functioning at hospital discharge, (2) mitigated muscle wasting, and (3) improved patient-reported outcomes, such as quality of life one month after hospital discharge.

## Methods

### Study design

This four-arm, multicentre RCT was registered in the Clinical Trials Registry (ChiCTR2200057685) and approved by the Fujian Provincial Hospital Human Research Ethics Committee (K2021-04-004). All procedures were carried out in compliance with ethical norms for human experimentation as well as the Helsinki Declaration of 1975 and its subsequent amendments. Written informed consent was obtained from all participants through an authorised surrogate decision-maker.

### Participants and settings

This trial was conducted in 10 ICUs at five academic and tertiary comprehensive hospitals in Fujian province, China. Medical centre enrolment criteria are more than 15 beds in each ICU, and more than 500 ICU patients admitted to each ICU per year. We included adult patients who were admitted to the ICU with medical critical illnesses.

### Inclusion criteria for patients were as follows

(1) aged 18 to 80 years, (2) expected to stay in the ICU for more than 48 h, (3) could walk independently two weeks before transfer to ICU, (4) APACHE-II ≥ 8 points, (5) patients were awake and able to cooperate with five standardised questions ≥ 3 points [21], and (6) patients or proxies signed written informed consent.

### Patients were excluded from the study if they met any of the following criteria

(1) incapable of doing the early activity or rehabilitation exercises, such as being in the acute phase of myocardial infarction, having a ruptured thoracic aortic aneurysm, or obstructive hypertrophic cardiomyopathy, uncontrolled lethal arrhythmia, pulmonary embolism, acute phase of asthma, severe pulmonary hypertension, myasthenia gravis, Guillain–Barré syndrome, recent deep vein thrombosis (DVT) or venous thromboembolism (VTE), active uncontrolled bleeding, restriction of activity during hospitalisation due to medical condition and other factors; (2) acute phase of brain injury and possible long-term physical dysfunction and impaired consciousness; cognitive dysfunction or mental impairment; (3) contraindication to enteral nutrition or the need for prolonged fasting; (4) inability to achieve muscle strength level 3 during ICU hospitalisation; (5) during femoral arterial cannulation.

### Patients were withdrawn if they met any of the following criteria

(1) voluntary withdrawal from the study during the intervention; (2) acute complications, making the continuation of the intervention impossible, or necessitating termination of the intervention due to changes in condition, death, etc.

### Randomisation and blinding

Participants who met the specified inclusion and exclusion criteria were assigned to one of four groups (RT group, HMB group, combination group, or control group receiving standard care) in a 1:1:1:1 ratio using a computer-generated random sequence. To ensure allocation concealment, each centre prepared sequentially coded, sealed, and opaque envelopes containing random numbers. Stratification was applied based on whether the participants received mechanical ventilation. The random sequence for each site was only accessible to the coordinator, ensuring blinding of physicians and study personnel regarding the hypothesis, group assignment, specific intervention protocols, and study endpoints. However, they were aware that a physical function intervention study was imminent.

Due to the inherent nature of RT and HMB, it was not possible to blind patients, families, or ICU clinicians in this study. However, to minimise bias, outcome assessments were performed by assessors and statisticians who were blinded to the study groups.

### Intervention protocol

#### Standard care

Participants in four groups received comprehensive rehabilitation and nutrition management, overseen by physicians and charge nurses. Early rehabilitation included activities such as passive joint mobilisation, passive sitting, bedside sitting, bicycle-assisted training, active bedside exercises, dynamic standing, assisted walking, or advanced muscular training, and so on. Dietary goals aimed for a total energy intake of 20–25 kcal/kg per day during the acute phase and 25–30 kcal/kg per day during the stable phase, with a target protein intake of 1.2–2 g/kg per day [22]. The attending physician decides on routine rehabilitative and dietary treatment for ICU patients, and these administrations were meticulously documented to account for complicating factors.

#### Resistance training (RT)

The intervention protocol encompassed three physical function levels: supine, sitting, and standing. Each level consisted of seven to eight actions, such as chest pressing, elbow flexion, rowing, ankle dorsiflexion, ankle plantarflexion, knee extension, hip flexion, bridge exercises, and abdominal breathing. These exercises targeted the upper and lower extremity muscles, as well as the core muscle groups. Visual illustrations of the training movements can be found in supplemental Figs. 1 and 2.

**Fig. 1:**
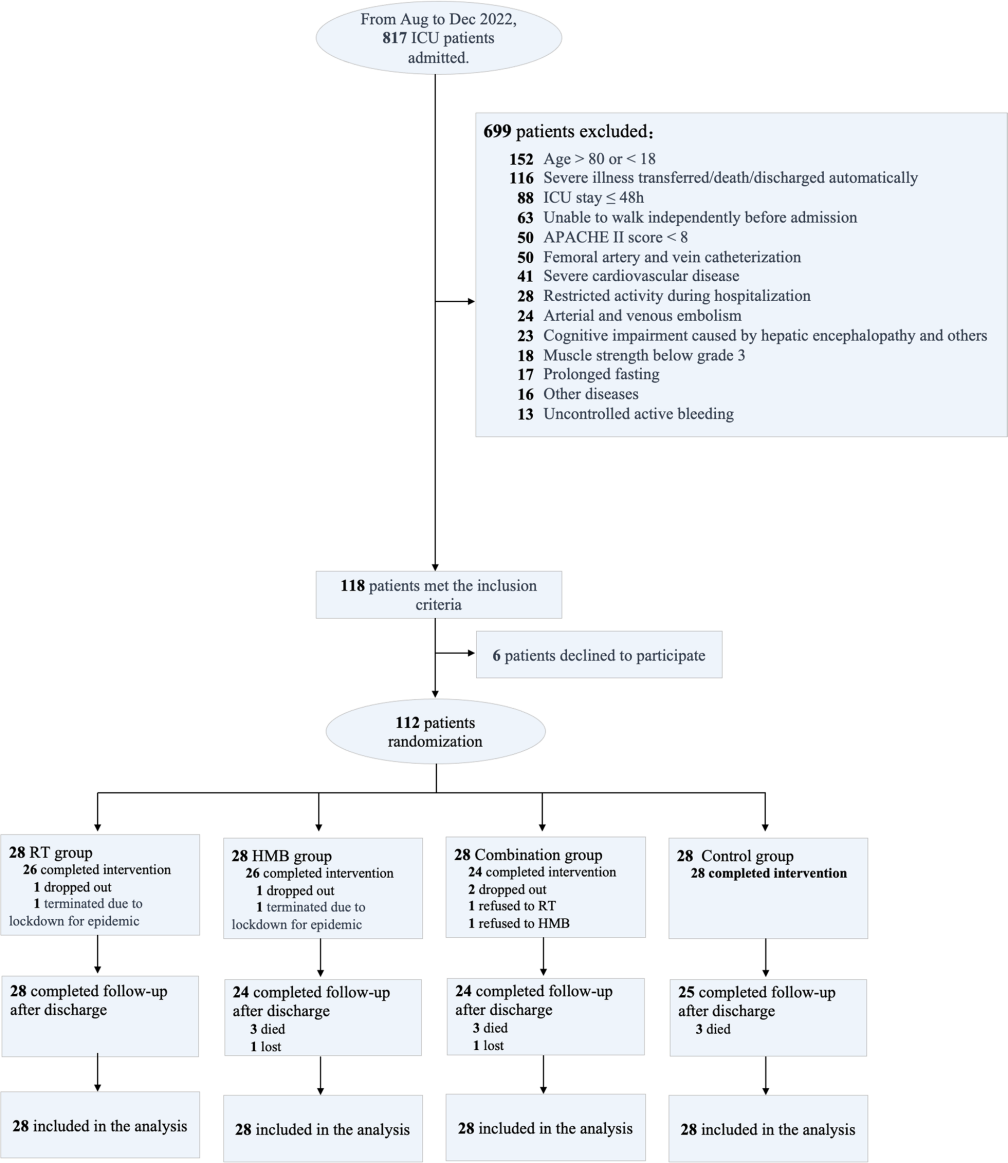
The flowchart of participants

**Fig. 2:**
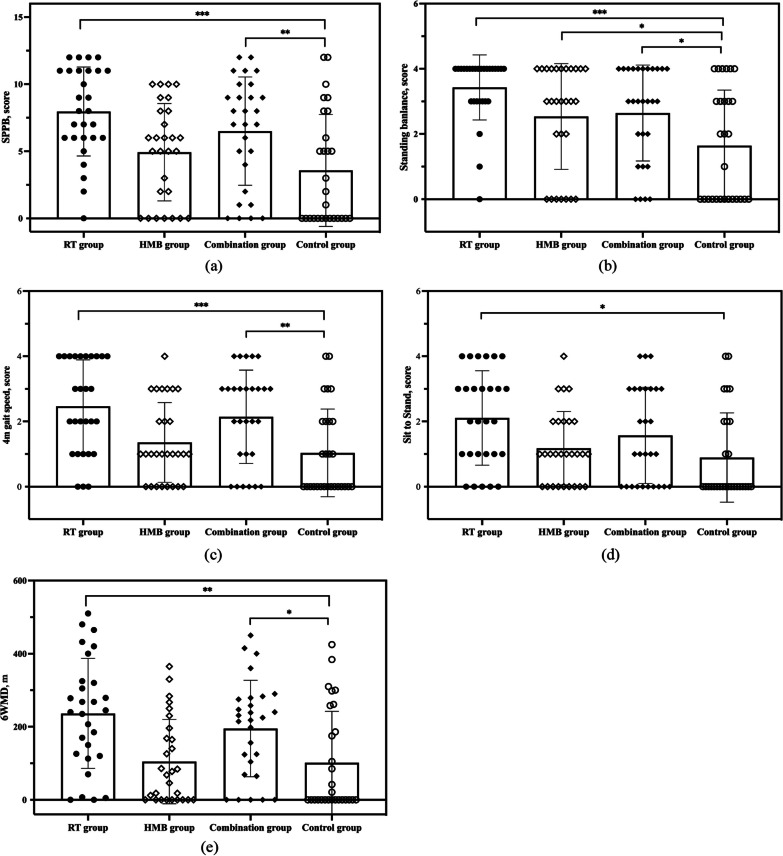
The results of primary outcomes among the four groups. (**a**) total score of Short Physical Performance Battery (SPPB); (**b**) standing balance score; (**c**) 4 m speed gait score; (**d**) sit to stand score; (**e**) 6-min walking distance (6MWD)

The RT intervention was conducted under the guidance of trained researchers, both in the ICU and in general ward, starting from randomisation until hospital discharge. Administered five times per week, each session lasted approximately 20 to 30 min and was comprised of warm-up, exercise, and cool-down phases. The exercises incorporated two levels of resistance: using body weight for limb movements and utilising TheraBand elastic bands (The Hygenic Corporation, Akron, OH, U.S.) with colour-coded levels of resistance (yellow and red).

#### HMB intervention

The nutritional supplement used in this study was JIROU (EnterNutr, Guangzhou), containing 8.32 g of maltodextrin, 1.5 g of HMBCa, 0.1 g of sweet orange powder, and 0.08 g of sucralose per 10 g. It provided 34 kcal of energy and no protein. The supplement, which included 1.5 g of HMB, was dissolved in 100 ml of warm water and administered orally or via tube feeding twice daily by nurses. HMB administration should commence when the patient is receiving enteral nutrition and should be discontinued if there is intolerance to enteral nutrition.

Furthermore, ensuring patient safety remained our paramount concern. Guided by clinical practice guidelines for early rehabilitation [23], we established specific criteria to initiate interventions. Real-time monitoring of adverse events was implemented by study personnel for the RT group, HMB group, and combination group, while retrospective chart reviews of nursing records were conducted for the control group. Adverse events encompassed various aspects, including: (1) cardiovascular events: hypotension (fluctuation > 20%, duration ≥ 1 min), hypertension, tachypnea, bradypnea, tachycardia, bradycardia, arrhythmia, chest discomfort, and dizziness; (2) accidental events: falls and dislodgement of tubes; (3) metabolic issues: hypoglycaemia; (4) patient complications: lower extremity deep vein thrombosis and pressure injuries; (5) gastrointestinal reactions: abdominal pain, diarrhoea, abdominal bloating, vomiting, and any other relevant events.

### Data collection and outcome measures

Outcome assessors were trained and masked to group allocation. Outcome measurements in this study were divided into four categories, that is, physical function, muscle strength, body composition, and health-related quality of life.

#### Physical function

(1) Short Physical Performance Battery (SPPB) was used to assess physical function, comprising a standing balance test, four-metre walking speed, and five sit-to-stand tests, each item worth 0–4 points, with a total score of 0–12 points [24].

(2) 6-Minute Walk Test (6MWT) To evaluate functional endurance capacity and mobility, participants were directed to walk independently along a 30-m hospital corridor and cover as much distance as possible within a six-minute duration [25]. The recorded outcome, denoted in meters, is known as the 6-minute walk distance (6MWD).

#### Muscle strength

(1) The MRC score was used to assess the muscle strength of the six major muscle groups of the extremities. Each group is divided into six levels according to the Oxford muscle strength scale, with a total score of 0–60. Higher scores represent greater muscle strength. MRC is considered the gold standard for the diagnosis of ICU-AW, and a score < 48 can be diagnosed as ICU-AW [26].

(2) Grip strength: The CAMRY EH101 handheld electronic grasp strength device was used to measure the voluntary contraction force of the dominant hand's musculature. The participant was placed in a supine, neutral forearm position [27, 28], elbow flexed 90 degrees, repeated three times, with the maximal value recorded.

#### Body composition

Bioelectrical impedance analysis (BIA, NUTRILAB, AKERN, Italy) was utilised to assess fat-free mass (FFM), appendicular skeletal muscle mass (ASMM), skeletal muscle index (SMI), and phase angle (PhA).

### Health-related quality of life

**(1) 36-Item Short-Form Health Survey** (**SF-36)** [29], which comprises 36 questions with the Physical Component Summary (PCS), and the Mental Component Summary (MCS).

**(2) Mini-Mental State Examination (MMSE)**, a simple tool for assessing cognitive function. A total of 30 items with 30 points is stratified by education, with illiterate groups ≤ 17 points, primary groups ≤ 20 points, and secondary and above groups ≤ 24 points deemed to have abnormal cognitive function [30].

**(3) Hospital Anxiety and Depression Scale (HADS)** consists of two subscales, anxiety and depression, with seven items each and 14 items in total, each of which is scored on a 0–3 scale, with a maximum score of 21 for each component of anxiety and depression, where a score of ≥ 8 is considered positive for anxiety or depressive symptoms [31].

**(4) Revised Impact of Event Scale (IES-R)** is a 22-item instrument that detects post-traumatic stress disorder (PTSD) symptoms [32]. It assesses the severity of avoidance, intrusion, and hyperarousal symptoms, the three categories of PTSD symptoms. The adoption of a threshold score of 33 out of 88 indicates severe psychological impact of a traumatic event.

6MWT and SPPB measurements were taken upon hospital discharge, recording a measurement of zero if a participant was unable to ambulate independently or complete the task. Body composition assessments were conducted at T0, T1w (1 week after intervention), T2w (two weeks after intervention), and hospital discharge. MRC score, grip strength, and MMSE were evaluated at T0, ICU discharge, and hospital discharge, excluding the aforementioned time points, and HADS were also conducted by telephone one month post-discharge. SF-36 and IES-R assessments were solely performed during the one-month follow-up visit. In cases where the individual had died by the one-month follow-up, SF-36 were recorded as zero.

### Primary outcomes

SPPB and 6WMD were measured at hospital discharge.

### Secondary outcomes

Assessments were conducted at each time point for body composition, MRC score, ICU-AW rate, grip strength, MMSE, HADS, SF-36, IES-R, and 60-day mortality, length of stay.

### Quality control

(1) A multicentre research team was established with sufficient personnel and reasonable hierarchy to ensure smooth project implementation. ICU nurse managers served as sub-centre leaders, while overall execution in each sub-centre was handled by head educators or quality control nurses from the respective departments. The intervention in this study was carried out by trained nurses who had substantial critical care or rehabilitation experience working in ICU settings. The eligibility criteria for intervention implementers and assessors in the sub-centres included: having a minimum of an associate degree, possessing over 10 years of ICU experience as nurses or rehabilitation therapists, demonstrating strong organisational and communication skills, and voluntary participation.

(2) Based on a preliminary feasibility study, a standardised research manual and action videos were created and uploaded to an online platform for the research team's reference. These nurses underwent a comprehensive one-month training programme facilitated by a multidisciplinary team. The training team included experienced research nurses as well as a physician specialised in rehabilitative medicine. The purpose of the training was to equip the nurses with the necessary knowledge and skills to effectively implement the study programme in the ICU environment. They were trained on the specific intervention techniques and protocols outlined in the study.

(3) To ensure the fidelity of the intervention, regular meetings were held with the intervention team. These meetings included quality control activities, such as reviewing intervention logs. The purpose of these reviews was to assess the competence of the interventionists in delivering the study programme and to address any questions or concerns that arose during the study period.

Overall, the training and quality control activities were essential in ensuring that the intervention was implemented consistently and accurately throughout the study.

### Statistical analysis

#### Sample size determination

A pre-study power analysis conducted using G Power software estimated that approximately 24 patients per group (with a total of 96) were required to detect a 35% improvement in physical function between the control and treatment groups, with a power of 0.8 and an alpha level of 0.05.

The data was processed using IBM SPSS software. All statistical tests were two-sided, with differences considered statistically significant when P < 0.05. The analysis followed the principles of intention-to-treat (ITT).

#### General characteristics analysis

Normality of continuous variables was assessed using the SW-test, PP-plot, and QQ-plot. Normally distributed variables were described using mean and standard deviation, while non-normally distributed variables were described using median and interquartile range. Categorical variables were described using frequency and percentage. Analysis of normally distributed continuous variables utilised the ANOVA test, while non-normally distributed variables were analysed using the Wilcoxon Rank Sum test. Categorical variables were analysed using the chi-square test or Fisher’s exact test, as appropriate. The missing characteristic values of one patient, including age, drinking habits, and NUTRIC score, were imputed with the mean values.

#### General linear mixed model

The GLMM investigated discrepancies in SPPB and 6WMD at hospital discharge, as well as SF-36 and IESR scores at one month post-discharge. It also explored differences and trends in FFM, ASMM, SMI, and PhA among the four groups at various assessment time points: T0, T1w, T2w, and hospital discharge. Additionally, the GLMM analysed variations and trends in MRC scores, ICU-AW, grip strength, and MMSE at T0, transfer out, and discharge. Furthermore, it compared differences in HADS, and FSS-ICU scores at T0, transfer out, discharge, and one month post-discharge among the four groups.

Fixed effects included group, time, and between group and time interactions to account for repeated measures. The random effect of the centre controlled for population heterogeneity across centres. Regression coefficients (β) with 95% confidence intervals were calculated to evaluate the impact of interventions—specifically RT, HMB supplementation, and their combination—compared to the control group. The results of body composition showed no missing values. Grip strength, MRC score, HADS, MMSE, SF-36, and IESR had missing values of less than 4%. Among the patients, 22.32% and 27.6% reported being unable to complete the SPPB and 6MWD assessments, respectively. As per prior criteria, their measurement values were recorded as 0. GLMM's capability to handle missing values eliminated the requirement for specific treatment of missing values in the concluding indicator section. Consequently, missing values for both primary and secondary outcome measures were not imputed in our study.

## Results

### Patient characteristics

A total of 817 patients from 5 centres were initially assessed for study eligibility from July 2022 to December 2022. Among them, 699 were excluded due to not meeting the inclusion and exclusion criteria, while six declined to participate. Consequently, 112 patients were randomly allocated. Of these, eight patients withdrew during the intervention, nine patients passed away after 60 days of enrollment, and two patients were lost to follow-up at one-month discharge. Therefore, a total of 101 patients (28 in the RT group, 24 in the HMB group, 24 in the combination group, and 25 in the control group) underwent follow-up testing. Thus, the intention-to-treat (ITT) analysis included a total of 112 patients, as shown in Fig. 1.

Five centres were included in this study, with patient numbers of 46 (41.1%), 25 (22.3%), 23 (20.5%), two (1.8%), and 16 (14.3%), respectively, and there were no statistically significant differences between the four arms (P > 0.05). The average age of all patients was 59.87 years, while the average ages of the four categories were 55.44, 62.68, 60.04, and 61.18 years, respectively, with no statistically significant differences (*P* > 0.05). The median APACHE II scores for all patients and the four groups were 16.0, 13.50, 15.0, 20.0, and 15.0, respectively, with no statistically significant differences (*P* > 0.05). There were no statistically significant differences between the median SOFA scores of the four groups (*P* > 0.05): 6.0, 6.0, 6.0, and 5.0. The most prevalent diagnoses were acute and chronic pulmonary diseases (46 cases, 41.1%), sepsis (25 cases, 22.3%), chronic kidney disease (7 cases, 6.3%), acute pancreatitis (8 cases, 7.1%), and other (26 cases, 23.1%). There were no statistically significant differences in the distribution of diagnoses between groups (*P* > 0.05). Table 1 presents several additional patient characteristics that show no statistical significance.

**Table 1:** Patient characteristics

Variables	All N = 112	RT group N = 28	HMB group N = 28	Combined group N = 28	Control group N = 28	Statistics	*P value*
Centre [N(%)]							
1	46(41.1)	11(39.3)	9(32.1)	4(50.0)	12(42.9)	7.272	0.839
2	25(22.3)	6(21.4)	6(21.4)	7(25.0)	6(21.4)		
3	23(20.5)	5(17.9)	6(21.4)	7(14.3)	8(28.6)		
4	2(1.8)	1(3.6)	1(3.6)	0(0)	0(0)		
5	16(14.3)	5(17.9)	6(21.4)	3(10.7)	2(7.1)		
Age [years]	59.87 ± 15.81	55.44 ± 15.63	62.68 ± 14.29	60.04 ± 14.95	61.18 ± 18.04	1.067	0.366
Male [N(%)]	88(78.57)	23(82.14)	23(82.14)	22(78.57)	20(71.43)	1.273	0.736
BMI [kg/m^2^, N (%)]	22.82 ± 4.20(rang13.67 to 38.74)	23.0 ± 3.92(rang 16.14 to 31.14)	23.01 ± 4.76(rang 14.79 to 38.74)	22.49 ± 3.19(rang 15.94 to 27.68)	22.77 ± 4.91(rang 13.67 to 33.27)	0.093	0.964
< 18.5	16(14.3)	4(14.3)	3(10.7)	3(10.7)	6(21.4)	4.158	0.901
18.5–24.9	65(58.0)	16(57.1)	17(60.7)	18(64.3)	14(50.0)		
25–29.9	25(22.3)	6(21.4)	6(21.4)	7(25.0)	6(21.4)		
≥ 30	6(5.4)	2(7.1)	2(7.1)	0(0)	2(7.1)		
Education [N(%)]							
Illiterate or semi-literate	21(18.8)	6(21.4)	6(21.4)	5(17.9)	4(14.3)	10.942	0.280
Elementary School	31(27.7)	6(21.4)	5(17.9)	8(28.6)	12(42.9)		
Junior to senior high school	46(41.1)	14(50.0)	13(46.4)	13(46.4)	6(21.4)		
College diploma or above	14(12.5)	2(7.1)	4(14.3)	2(7.1)	6(21.4)		
Work prior to admission							
None	12(10.7)	2(7.1)	2(7.1)	6(21.4)	2(7.1)	7.693	0.261
Physical labour	29(25.9)	10(35.7)	5(17.9)	5(17.9)	9(32.1)		
Non-physical labour	71(63.4)	16(57.1)	21(75.0)	17(60.7)	17(60.7)		
Smoking [N(%)]	48(42.9)	11(39.3)	15(53.6)	11(39.3)	11(39.3)	1.750	0.626
Drinking [N(%)]	46(41.4)	16(57.1)	11(39.3)	10(35.7)	9(33.3)	4.008	0.261
Length of stay prior to ICU [days, M(Q_25_,Q_75_)]	0(0,2.0)	0(0,1.0)	0(0,2.75)	0.5(0,3.0)	0(0,2.0)	2.667	0.446
ICU Patient Sources [N(%)]							
Emergency	54(48.2)	13(46.4)	14(50.0)	8(28.6)	19(67.9)	12.319	0.196
General ward	30(26.8)	6(21.4)	8(28.6)	11(39.3)	5(17.9)		
Post-operation	4(3.6)	2(7.1)	0(0)	2(7.1)	0(0)		
Transferred from another hospital	24(21.4)	7(25.0)	6(21.4)	7(25.0)	4(14.3)		
Plan to be transferred to ICU [N(%)]	7(6.3)	4(14.3)	0(0)	2(7.1)	1(3.6)	5.333	0.149
Diagnosis [N(%)]							
Acute and chronic lung diseases	46(41.1)	7(25.0)	11(39.3)	11(39.3)	14(60.7)	14.164	0.290
Sepsis	25(22.3)	7(25.0)	8(28.6)	6(21.4)	4(14.3)		
Chronic kidney disease	7(6.3)	3(10.7)	0(0)	2(7.1)	2(7.1)		
Acute Pancreatitis	8(7.1)	4(14.3)	1(3.6)	1(3.6)	2(7.1)		
Others	26(23.2)	7(25.0)	8(28.6)	8(28.6)	3(10.7)		
Complications [N(%)]							
Respiratory failure	55(49.1)	9(32.1)	16(57.1)	13(46.4)	17(60.7)	5.537	0.136
Heart failure	19(17.0)	2(7.1)	7(25.0)	4(14.3)	6(21.4)	3.740	0.291
Sepsis	30(26.8%	7(25.0)	9(32.1)	7(25.0)	7(25.0)	0.546	0.909
Multiple organ failure	8(7.1)	0(0)	5(17.9)	1(3.6)	2(7.1)	7.538	0.057
Charlson Comorbidity Index [M(Q_25_,Q_75_)]	3.0(2.0,5.0)	2.0(2.0,3.75)	3.5(2.0,5.0)	4.0(2.0,5.0)	3.0(3.0,5.0)	6.082	0.108
Oxygenation index at admission [M(Q_25_,Q_75_)]	245.0(178.0,317.50)	238.0(179.0,363.25)	281.50(168.25,306.25)	245.0(177.25,307.50)	246.0(182.25,315.0)	0.242	0.971
APACHEII [M (Q_25_, Q_75_)]	16.0(12.0,23.0)	13.50(9.50,21.75)	15.0(10.25,20.50)	20.0(15.25,25.75)	15.0(12.25,22.75)	7.697	0.053
SOFA [M (Q_25_, Q_75_)]	6.0(4.0,8.0)	6.0(3.25,7.0)	6.0(2.50,9.75)	6.0(4.0,8.75)	5.0(4.0,6.0)	0.917	0.821
NRS2002[M (Q_25_, Q_75_)]	3.0(3.0,4.0)	3.0(3.0,4.0)	3.0(3.0,4.75)	4.0(3.0,4.0)	4.0(3.0,4.0)	1.940	0.585
NUTRIC	4.32 ± 1.98	3.78 ± 1.95	4.43 ± 1.81	4.86 ± 2.00	4.19 ± 2.06	1.464	0.229
Length of ICU stay [days, M (Q_25_, Q_75_)]	6.50(4.0,12.0)	5.0(3.0,9.75)	9.0(4.0,17.75)	6.0(3.0,9.0)	7.5(5.0,14.0)	7.430	0.059
Endotracheal intubation [N(%)]	34(30.9)	9(32.1)	8(30.8)	8(28.6)	9(32.1)	0.112	0.990
Duration of mechanical ventilation [h, M (Q_25_, Q_75_)]	0(0,41.0)	0(0,7.0)	0(0,30.0)	0(0,34.75)	0(0,116.75)	2.046	0.563
60-Day Mortality [N(%)]	9(8.0)	0(0)	3(10.7)	3(10.7)	3(10.7)	3.262	0.353
Length of hospital stay [days, M (Q_25_, Q_75_)]	15.0(11.0,27.25)	14.0(9.25,19.50)	21.0(11.25,34.75)	15.50(13,23.75)	14.0(10.0,36.50)	5.155	0.161
Length of hospital stay after ICU [days, M (Q_25_, Q_75_)]	14.0(9.0,22.75)	13.0(8.25,19.25)	13.5(10.25,24.25)	14.0(10.50,22.25)	13.50(7.25,28.25)	1.976	0.577
Duration of ICU stay prior to the study [days, M (Q_25_, Q_75_)]	2.0(2.0,5.0)	2.0(1.0,7.0)	3.0(2.0,8.0)	2.0(1.25,3.0)	2.50(2.0,5.75)	5.046	0.168

BMI: Body Mass Index = Weight / Height^2 (kg/m^2); MODS: Multiple Organ Dysfunction Syndrome; APACHE II: Acute Physiology and Chronic Health Evaluation II; SOFA: Sequential Organ Failure Assessment; NRS2002: Nutritional Risk Screening 2002; NUTRIC: Nutrition Risk in Critically Ill; CCI: Charlson Comorbidity Index

### Delivery and adherence to the interventions

The median duration of ICU stay prior to the study had no difference among four groups, as illustrated in Table 1. The actual frequency of intervention in the RT group and the combined group was 8.0 and 9.5 sessions, respectively. The respective median compliance rates were 90.60% and 100.0%, respectively. The median duration per intervention was 23.7 min and 20.0 min, respectively. In the RT group, the median proportions of patients using elastic bands, yellow bands, and red bands for training were 66.67% (25.0%, 88.19%), 25.0% (0, 44.65%), and 0 (0, 49.58%), respectively. In the combined group, the proportions were 76.39% (29.22%, 87.85%), 40.0% (0, 81.88%), and 0 (0, 35.18%), respectively. 5.42% (13.49%, 58.48%) of patients in the RT group performed supine exercises, while 37.5% (22.31%, 55.95%) performed seated exercises, and 25% (0%, 35%) performed standing exercises. In the combined group, the proportions were 47.22% (32.81%, 89.29%), 23.37% (0%, 56.25%), and 0% (0%, 18.40%), respectively. In the ICU, the RT group had a proportion of 0% (0%, 62.6%) for elastic band usage, whereas the combination group had a proportion of 54.5% (0%, 100%). In the general ward, both groups exhibited a proportion of 77.5% (17.5%, 100%) and 76.4% (50%, 100%), respectively. The cumulative number of incomplete sessions was 28 (10.98%) and 46 (14.56%), with reported weakness being the most common reason. No adverse events occurred during the training process. See supplementary Table 1.

The actual number of interventions conducted by the HMB group and the combination group were 22.0 and 18.0, with respective compliance rates of 91.22% and 92.03%, respectively. The cumulative number of incomplete interventions was 70 (9.3%) and 93 (13.2%) for the two groups, respectively. The main cause, which accounted for 25 (35.71%) and 32 (34.40%) occurrences, respectively, was patient miss taking, especially in general wards. There were 15 (21.43%) and 14 (15.05%) occurrences of temporary discontinuance as a result of gastrointestinal problems, respectively. In the RT group, there were no confirmed occurrences of gastrointestinal responses associated with HMB, and 36 (51.43%) incidents were found to be unrelated after discussion and analysis with the attending physician. There were 50 (53.76%) cases where it was found that the gastrointestinal reactions to HMB were unrelated, while there were no confirmed cases of gastrointestinal reactions in the combination group. Please see supplementary Table 2 for more information.

### Daily activity and energy intake

There were no statistically significant differences between the four groups in terms of gastrointestinal reactions, average daily sitting time, walking time, cumulative energy deficiency, cumulative protein deficiency, or delirium (*P* > 0.05), as shown in Table 2.

**Table 2:** The differences in daily profile among the four groups

	RT group	HMB group	Combination group	Control group	statistics	*P* value
Adverse gastrointestinal effects N(%)	6(21.4)	5(17.9)	4(14.3)	4(14.3)	.697	0.874
Average daily sitting time, min	19.33(7.68,44.81)	20.0(0,58.34)	25.0(3.75,76.25)	29.28(0,54.31)	0.639	0.887
Average daily walking time, min	15.9(5,23.09)	8.75(0,23.83)	9.58(0,26.87)	5.73(0,20.5)	2.191	0.534
Accumulated energy deficiency, kcal	−5086.4(−8246.75,−1903.65)	−4944.50(−8651,−1848.5)	−5168.0(−8663,−2488.3)	−3449.5(−5010.75,148.55)	3.573	0.311
Accumulated protein deficiency, g	−255(−478.23,−92.37)	−279.63(−403.54,−120.7)	−282.54(−371.07,−135.69)	−267.63(−374,−121.8)	0.235	0.972
The average energy intake of enteral nutrition in the ICU, kcal	574.75(322.13,889.88)	797.5(593.87,1205)	712.63(290.88,1059.97)	745.25(494.38,1132.46)	4.101	0.251
The average energy intake of parenteral nutrition in the ICU, kcal	0(0,0)	0(0,0)	0(0,142.14)	0(0,0)	4.954	0.175
The average energy intake of enteral nutrition in the general ward, kcal	735.5(491.25,1203.71)	970.65(667.74,1117.88)	797.96(561.22,1039.14)	777.44(532.44,1213.49)	2.244	0.523
The average energy intake of parenteral nutrition in the general ward, kcal	0(0,7.43)	0(0,0)	0(0,0)	0(0,0)	0.708	0.871
The average protein intake of enteral nutrition in the ICU, g	24(10,40.11)	37.38(22,54.4)	29(9.8,46.54)	30.97(18.19,42.29)	7.038	0.071
The average protein intake of parenteral nutrition in the ICU, g	0(0,0)	0(0,0)	0(0,2.2)	0(0,0)	2.324	0.508
The average protein intake of enteral nutrition in the general ward, g	36.71(22,53.22)	43.58(31.32,57.48)	39.86(24.61,55)	31.6(21.36,40.25)	6.289	0.098
The average protein intake of parenteral nutrition in the general ward, g	0(0,0)	0(0,0)	0(0,0)	0(0,0)	0.693	0.875
Delirium [N(%)]	3(10.07)	4(14.30)	3(10.07)	3(10.07)	0.261	0.967

### Primary outcomes

GLMM analysis revealed significant differences between the four groups in terms of SPPB overall score, balance ability, four-metre gait speed score, and 6MWD (*P* < 0.001). The SPPB dimension-specific sit-to-stand score was not statistically significant (*P* > 0.05). Compared with the control group, the RT group and combination group demonstrated significant improvements in SPPB score (effect sizes β values were 3.848 [95%CI: 1.827–5.870] and 2.832 [95%CI: 0.812–4.853], *P* < 0.001 and *P* < 0.01), and 6MWD (β values were 99.768 [95%CI: 30.741–168.794] and 88.577 [95%CI: 19.569–157.584], respectively, *P* < 0.01 and *P* < 0.05). but the HMB group was not significant (*P* > 0.05), see Table 3 and Fig. 2.

**Table 3:** Results of GLLM analysis for comparison of SPPB/6MWD/SF-36/IESR

Outcomes	$\mathrm{RT group}$ HMB group			Combination group		Control group		*F*	*P*
	(x ± s)	β(95%CI)	(x ± s)	β(95%CI)	(x ± s)	β(95%CI)	(x ± s)		
SPPB score	7.5 ± 3.48	3.848(1.827–5.870)^***^	4.93 ± 3.63	1.298(-0.727–3.322)	6.50 ± 4.03	2.832(0.812–4.853)^**^	3.57 ± 4.18	5.522	**0.001**
Balance	3.32 ± 1.02	1.614(0.839,2.388)^***^	2.56 ± 1.62	0.828(0.052,1.603)^*^	2.64 ± 1.47	0.947(0.173,1.721)^*^	1.643 ± 1.70	5.749	**0.001**
4 m gait speed	2.43 ± 1.48	1.39(0.663,2.116)^***^	1.36 ± 1.22	0.322(-0.405,1.049)	2.14 ± 1.43	1.1(0.373,1.826)^**^	1.04 ± 1.35	6.296	**0.001**
Sit to stand	1.71 ± 1.384	0.786(0.086,1.486)^*^	1.18 ± 1.12	0.271(-0.43,0.973)	1.57 ± 1.48	0.618(-0.081,1.317)	0.89 ± 1.37	1.993	0.119
6WMD, m	204.81 ± 136.35	99.768(30.741–168.794)^**^	104.64 ± 115.4676	0.690(-68.453–69.833)	195.17 ± 131.83	88.577(19.569–157.584)^*^	101.79 ± 140.53	4.884	**0.003**
SF-36 score	571.57 ± 152.71	132.861(23.161 ± 242.562)^*^	493.23 ± 210.29	54.991(-54.883 ± 164.864)	486.57 ± 235.28	46.576(-63.132 ± 156.283)	431.11 ± 223.94	1.995	0.119
PCS	246.18 ± 74.01	64.525(13.538 ± 115.512)	209.07 ± 94.76	27.517(-23.923 ± 78.957)	194.89 ± 106.52	13.35(-37.654 ± 64.354)	180.48 ± 103.94	2.360	0.076
MCS	292.35 ± 76.88	58.36(4.622 ± 112.098)	257.67 ± 107.13	25.965(-28.217 ± 80.147)	256.86 ± 120.9	20.796(-32.867 ± 74.460)	225.63 ± 111.92	1.596	0.195
IESR score	21.93 ± 8.05	3.589(-1.104 ± 8.282)	22.23 ± 8.15	3.553(-1.324 ± 8.429)	25.46 ± 8.31	6.492(1.626 ± 11.358)	23.17 ± 7.35	0.995	0.399
Avoidance	8.18 ± 3.58	-37.544(-89.903 ± 14.815)	7.88 ± 2.72	-6.73(-61.118 ± 47.658)	9.42 ± 3.5	-37.491(-91.795 ± 16.813)	8.38 ± 3.32	0.990	0.401
Intrusion	7.89 ± 2.77	0.304(-1.391 ± 1.998)	8.29 ± 2.94	0.625(-1.134 ± 2.384)	9.13 ± 2.56	1.125(-0.634 ± 2.884)	8.38 ± 2.26	0.955	0.417
Hyperarousal	5.89 ± 2.22	-0.042(-1.758 ± 1.675)	6.54 ± 2.96	0.833(-0.948 ± 2.615)	7.25 ± 3.83	1.167(-0.615 ± 2.948)	6.5 ± 2.09	1.047	0.375

SPPB: Short Physical Performance Battery; 6WMD: Six-Minute Walk Distance; SF-36: Short-Form-36 Health Survey; PCS: Physical Component Summary; MCS: Mental Component Summary; IES-R: Impact of Event Scale-Revised

### Secondary outcomes

#### 60-day mortality and length of stay

This study observed a 60-day mortality rate of 8.0%, with no cases in the RT group, and 10.7% (three cases) in each of the other three groups. The difference was not statistically significant (P > 0.05). Additionally, there were no significant differences (*P* > 0.05) in the length of hospital stay, ICU stay, and post-ICU stay among all study subjects. Please refer to Table 1 for specific details.

#### Body composition

The results of the GLMM analysis indicated there were no statistically significant differences (*P* > 0.05) in the group, time, and the group and time interaction effects of FFM, ASMM, SMI, and PhA. See supplementary Table 3.

#### Muscle strength

**Fig. 3:**
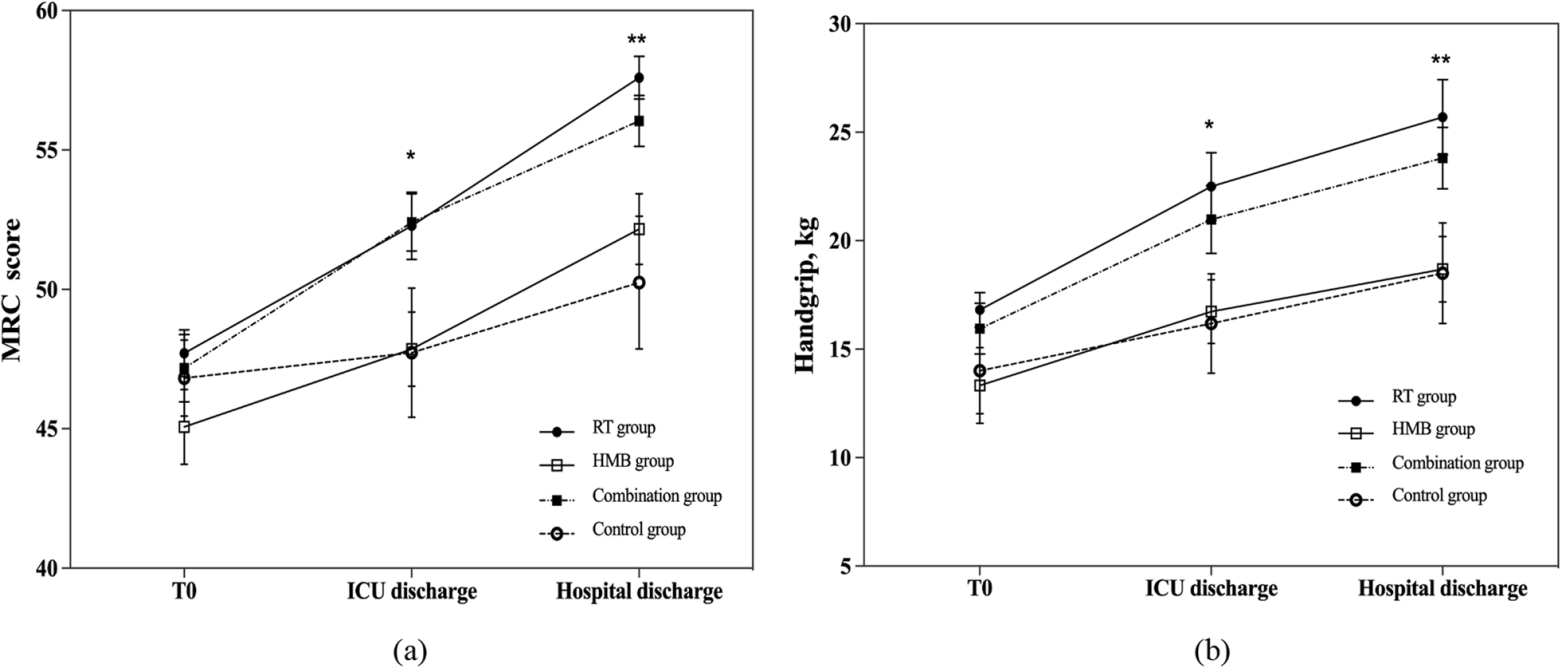
The trajectory of changes in muscle strength at different time points. **a** MRC score; **b** handgrip strength

Based on the results of the GLMM main effect analysis, significant differences were observed between groups and over time for MRC scores, ICU-AW, and grip strength (*P* < 0.05). However, the group and time interaction effects were not statistically significant (*P*  > 0.05). Both the RT group and the combined group showed significantly higher MRC scores than the control group (*P*  < 0.05), with respective β values of 4.724 (95%CI:0.421–9.027) and 4.819 (95%CI:0.516–9.121) upon ICU discharge, and 7.519 (95%CI:3.424–11.614) and 5.926 (95%CI:1.832–10.020) on hospital discharge. Grip strength values in the RT group and the combined group were also significantly higher than in the control group (*P*  < 0.05), with respective β values of 6.254 (95%CI:1.435–11.073) and 4.873 (95%CI:0.056–9.691) upon ICU discharge, and 7.123 (95%CI:2.221–12.025) and 5.373 (95%CI:0.473–10.274) upon hospital discharge. The effect sizes at hospital discharge were consistently greater than those at ICU discharge. No statistically significant differences were found in the HMB group. See Table 4 and Fig. 3.

**Table 4:** The results of GLLM analysis for muscle strength comparison among the four groups

Outcomes	T0	ICU discharge	Hospital discharge		
	$(\overline{x }\pm s)$	$(\overline{x }\pm s)$	*β(95%CI)*	$(\overline{x }\pm s)$	*β(95%CI)*
MRC score^a^					
RT group	47.71 ± 4.42	52.28 ± 6.40	4.724(0.421–9.027)^*^	57.59 ± 4.09	7.519(3.424–11.614)^***^
HMB group	45.07 ± 7.09	47.86 ± 7.09	0.288(-4.017–4.593)	52.16 ± 6.73	2.074(-2.024–6.171)
Combination group	47.17 ± 6.38	52.41 ± 5.45	4.819(0.516–9.121)^*^	56.04 ± 4.87	5.926(1.832–10.020)^**^
Control group	46.82 ± 7.17	47.73 ± 12.29	Reference	50.25 ± 12.6	Reference
*F*	0.90	2.992		5.505	
*P*	0.441	0.031		0.001	
ICU-AW^b^					
RT group	14(50.0)	8(28.6)	0.182(-0.091–0.455)	2(7.1)	0.245(-0.049–0.538)
HMB group	17(60.7)	15(53.6)	−0.092(−0.349–0.165)	9(32.1)	−0.072(−0.362–0.219)
Combination group	18(64.3)	7(25.0)	0.191(−0.081–0.463)	3(10.7)	0.169(−0.114–0.452)
Control group	14(50.0)	11(39.3)	Reference	6(21.4)	Reference
*F*	0.548	2.191		1.633	
*P*	0.650	0.089		0.182	
Handgrip^c^					
RT group	16.81 ± 4.21	22.50 ± 8.23	6.254(1.435–11.073)^*^	25.7 ± 9.20	7.123(2.221–12.025)^**^
HMB group	13.33 ± 9.20	16.74 ± 7.81	0.446(−4.423–5.315)	18.68 ± 8.01	0.061(−4.892–5.014)
Combination group	15.95 ± 6.20	20.98 ± 8.24	4.873(0.056–9.691)^*^	23.81 ± 7.49	5.373(0.473–10.274)^*^
Control group	14.01 ± 10.48	16.18 ± 12.10	Reference	18.5 ± 12.28	Reference
*F*	1.228	3.267		4.286	
*P*	0.299	0.022		0.006	

aMRC model: The Medical Research Council score, group: *F* = 7.95, *P* < 0.001, time: *F* = 29.824, *P* < 0.001, group × time interaction: *F* = 1.251, *P* = 0.28;

bICU-AW model: Intensive care unit-acquired weakness, defined as MRC < 48, group: *F* = 3.272, *P* = 0.021, time: *F* = 16.433, *P* < 0.001, group × time interaction: *F* = 0.993, *P* = 0.430

cHandgrip model: group: *F* = 8.385, *P* < 0.001, time: *F* = 17.282, *P* < 0.001, group × time interaction: *F* = 0.468, *P* = 0.832;

^*^P < 0.05, **P < 0.01, ***P < 0.00

#### Health-related quality of life

There were no differences (*P* > 0.05) in HADS-A, HADS-D, and MMSE at any time point. By the one-month follow-up visit, the differences among the four groups in terms of SF-36 score, PCS, MCS, and IESR were not statistically significant (*P* > 0.05). See supplementary Table 4.

## Discussion

Our findings indicate that RT improved physical function, muscle strength, and physical activity in MICU patients to a comparable extent to the combined intervention group. However, neither RT, HMB intervention, nor the combined intervention demonstrated efficacy in reducing muscle wasting or enhancing patients’ self-reported quality of life at one month post-discharge. Our study is groundbreaking in that it is the first ever multicentre randomised controlled trial with four arms to demonstrate that RT with or without HMB intervention can effectively enhance physical function in MICU survivors. The intervention encompasses the timeframe from ICU admission to patient transfer and discharge. Its continuum throughout the hospitalisation process optimally meets the rehabilitation requirements of MICU patients, highlighting another aspect of its novelty.

The utilisation of combined exercise and nutritional interventions in the ICU population remains limited, despite evidence from RCTs conducted in recent years. These trials have shown that integrating nutrition and exercise interventions can improve muscle mass, muscle strength, and physical function in critically ill patients [33–35]. However, divergent outcomes observed in our research may be attributed to variations in the design and execution of these clinical trials. The three RCT studies [33–35] we reviewed employed a two-arm design, comparing the efficacy of high-protein nutritional intervention combined with either neuromuscular electrical stimulation or in-bed cycle ergometry exercise, compared to a control group receiving usual care. Due to inherent limitations in the study design, it remains inconclusive as to whether the observed intervention effects can be ascribed to exercise intervention, high-protein intervention, or their synergistic effects. To address this, a four-group trial would be the optimal approach to evaluate the reciprocal benefits of a combined intervention involving nutrition and exercise [36]. Our study employed a four-group research design and applied a GLLM analysis. Findings revealed that the RT and combined intervention groups exhibited substantial gains in SPPB/6MWD, MRC scores, and grip strength compared to the control group, with similar effect sizes. However, the difference in HMB did not reach statistical significance.

The lack of statistically significant differences in body composition (FFM, SMI, ASMM, and PhA) and physical function (SPPB/6MWD, MRC, grip strength, etc.) associated with HMB intervention may serve as an additional important factor explaining the absence of advantages in the combined intervention of this study. Our findings align with prior research by Viana et al. [12] and Supinski et al. [13], which indicate that a 10-day intervention of 3 g HMB per day does not yield increases in quadriceps muscle thickness and strength. However, Nakamura et al. [11], through a subgroup analysis of patients with SOFA scores < 10, observed that a mixed supplement comprising 3 g HMB, 14 g arginine, and 14 g glutamine significantly mitigates decline in femoral muscle volume. There are several potential reasons that may explain the findings of this study. First, compared to other clinical populations, ICU patients are in a state of heightened catabolic metabolism, characterised by decreased anabolic hormone synthesis and increased catabolic hormone release [37]. The effects of HMB may not be sufficient to counteract the muscle wasting observed in ICU patients. Second, ICU patients often have impaired gastrointestinal function and reduced absorptive capacity, which may impede the intermediate processes of HMB ingestion, absorption, and efficacy. In our study, a total of 19 cases (16.96%) experienced gastrointestinal intolerance, such as abdominal distension and diarrhoea, which affected the absorption of nutritional supplements. Last, the efficacy of interventions by Nicolaas et al. [38] and Nakamura et al. [11] also provides a potential explanation for this study. These studies not only supplemented HMB, but also included protein or essential amino acids. Since HMB itself does not provide energy or protein, but rather promotes muscle protein synthesis and reduces degradation [6, 7], its optimal effects may require sufficient substrate support. In our study, the HMB group experienced an average cumulative energy loss of −4,944.50 kcal and an average cumulative protein loss of −279.63 g during hospitalisation, indicating a state of malnutrition across all groups. This suggests the need for further exploration of the potential synergistic effects of high-protein supplementation in combination with HMB.

In our study, we observed significant improvements in SPPB scores, 6MWD MRC score, and grip strength in both the RT group and the combined group. The effectiveness of these enhancements can be attributed to the meticulous specification of our research protocol. Notably, this is the first study to implement a stratified RT intervention for ICU patients, which includes supine, sitting, or standing positions based on individual functional capacity. Our interventions encompass physical functional training alongside muscle strength training, yielding more pronounced effects [39]. Additionally, specialised movements, such as elbow flexion, chest press, and rowing, were employed to enhance upper limb muscular strength, whereas exercises such as sitting-to-standing and straight leg lifts targeted lower limb muscle strength. The incorporation of diaphragmatic breathing during each exercise also contributed to improved cardiopulmonary function. As a result, the RT intervention significantly enhanced physical function, walking abilities, and muscle strength. When comparing our study's findings with those of Morris et al.'s [19] related research, slight disparities emerge. Morris et al. implemented a standardised intervention protocol encompassing passive joint movement, physical therapy, and elastic band resistance training for mechanically ventilated patients experiencing acute respiratory failure from ICU admission until hospital discharge. The mean SPPB scores at discharge were reported as 4.7, displaying no notable distinction. Nonetheless, disparities exist between our methodologies, which may account for the incongruent outcomes. In Morris et al.'s study, elastic band resistance training constituted only a fraction—specifically, 36%—of the intervention. Conversely, our study adopted a progressive approach to resistance, commencing with bodyweight exercises and gradually incorporating yellow and red elastic bands. Notably, both groups engaged in over 60% of the elastic band exercises, likely contributing to the superior post-discharge functional outcomes observed.

This research has several limitations. Firstly, generalising our study findings to ICU patients with higher disease severity is limited due to potential selection bias in our sample. This is evident from the low proportion of mechanically ventilated patients (30.9% of participants) and low to intermediate disease severity, as indicated by a median SOFA score of 6.0, a median APACHE II score of 16.0 and a median length of ICU stays of 6.5 days. Insufficient sample size hindered meaningful subgroup analysis. As a result, the effectiveness of the intervention for patients with higher disease severity upon admission, who are at the highest risk for ICU-AW, remains inconclusive. Future research with larger samples and stratified analyses should determine the efficacy of this intervention protocol in different disease severities. Second, although the reasons why the combined intervention did not outperform the individual intervention are extensively discussed in this study, we observed lower survival and compliance rates in the combined intervention group during the intervention process. However, we did not conduct a comprehensive investigation to ascertain whether this was due to population heterogeneity, or substantial barriers associated with the combined intervention. Last, the study subjects had to simultaneously meet the requirements for resistance training (being awake, able to actively cooperate, and a muscle strength level of at least grade 3) and the conditions for HMB intervention (initiating enteral nutrition) to receive the intervention. This may have caused a delay in the intervention's scheduling, thereby influencing the outcomes.

## Conclusion

This novel multicentre four-arm RCT has shown that multilevel RT intervention, with or without HMB intervention throughout the entire hospitalisation period, seems to enhance the physical function and muscle strength of MICU patients. However, none of these treatments had a significant impact on body composition or quality of life, based on health reports. Further investigations are necessary to validate these findings and explore potential implementation strategies.

### Supplementary Information

**Additional file 1.** 1. Overview of Resistance Training Intervention Program. 2. Examples of Resistance Training Exercises. 3. Resistance training intervention profile. 4. HMB intervention profile. 5. The results of secondary outcomes related to body composition. 6. The results of secondary outcomes related to psychological and cognitive function.

## Abbreviations

6MWD: 6-Minute walk distance
ASMM: Appendicular skeletal muscle mass
FFM: Fat-free mass
GLMM: General linear mixed model
HADS: Hospital anxiety and depression scale
HMB: Beta-hydroxy-beta-methylbutyrate
IES-R: Revised impact of event scale
ICU-AW: ICU-acquired weakness
MMSE: Mini-mental state examination
MRC: The Medical Research Council Score
PhA: Phase angle
RT: Resistance training
SF-36: 36-Item short-form health survey
SPPB: Short physical performance battery
SMI: Skeletal muscle index

## Declarations

### Availability of data and materials

The datasets generated and/or analysed during the proposed study will be available from the corresponding author upon reasonable request.
