# Supplementary material for: Effects of a multilevel intervention of resistance training with or without beta-hydroxy-beta-methylbutyrate in medical ICU patients during entire hospitalisation: a four-arm multicentre randomised controlled trial
Source: Crit Care. 2023 Dec 15;27:493. doi: 10.1186/s13054-023-04698-x (PMC10724983; doi:10.1186/s13054-023-04698-x)
Supplement: Supplementary file 1 — Additional file 1. 1. Overview of Resistance Training Intervention Program. 2. Examples of Resistance Training Exercises. 3. Resistance training intervention profile. 4. HMB intervention profile. 5. The results of secondary outcomes related to body composition. 6. The results of secondary outcomes related to psychological and cognitive function. [file 13054_2023_4698_MOESM1_ESM.docx]

**Supplementary**


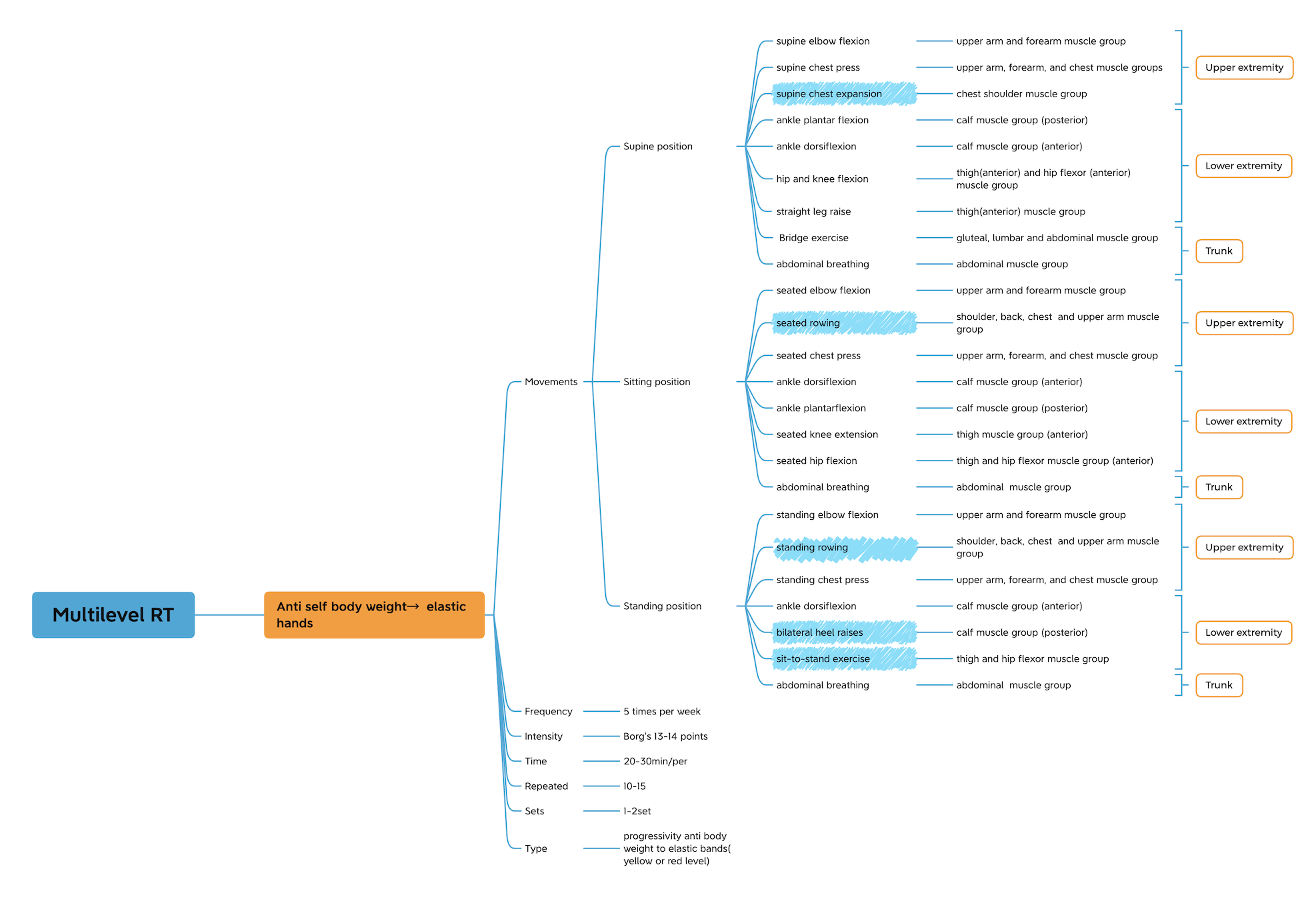


Figure 1. The intervention protocol overview


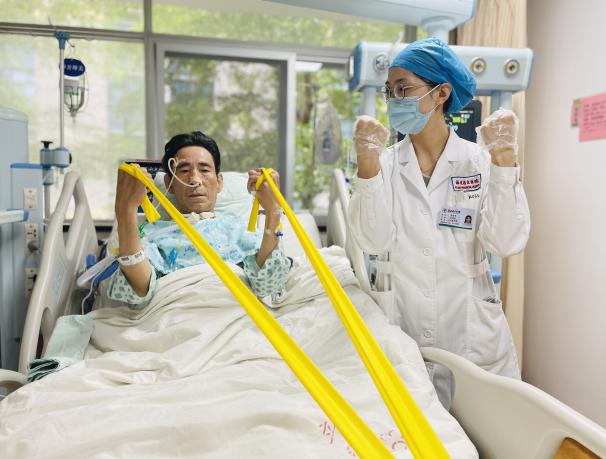

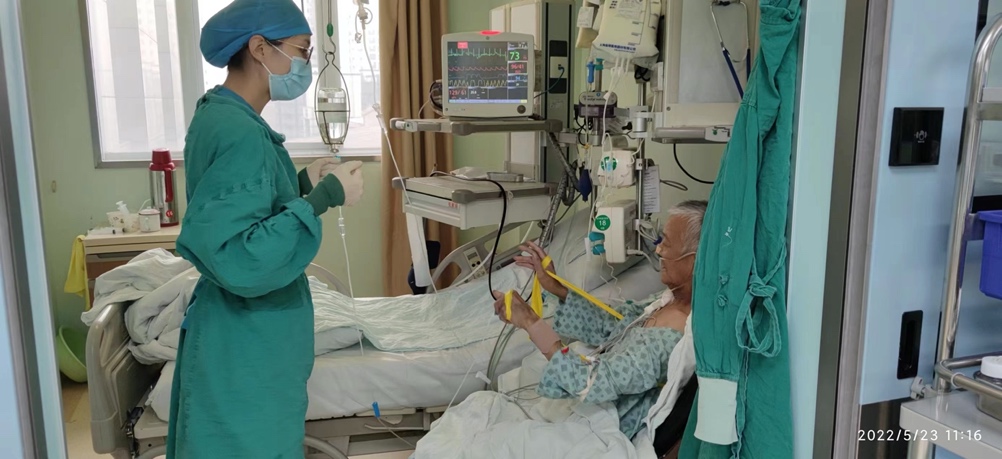


(a) (b)


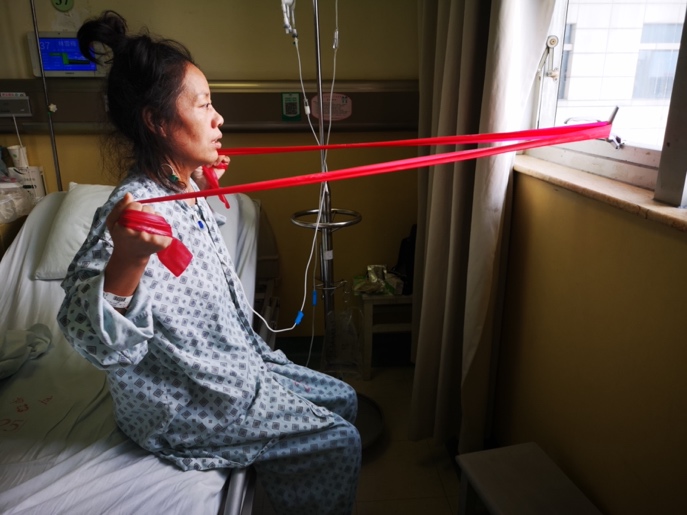

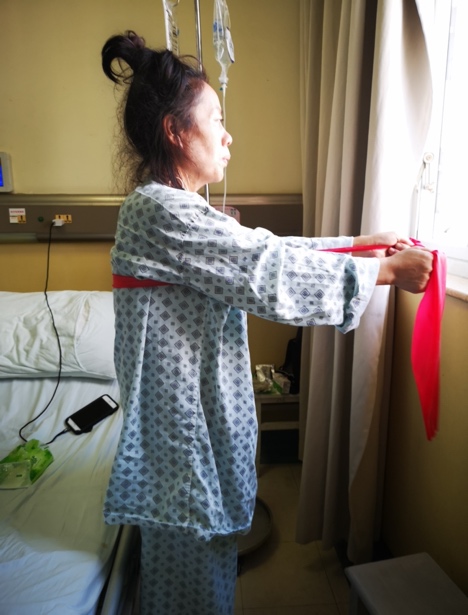


(c)

Figure 2 Examples of exercise movements. (a) Elbow flexion in supine position employing a yellow elastic band in ICU. In supine or semi-reclined position, the individual conducts elbow flexion exercises with the aid of an elastic band. The elastic band is looped around the foot of the bed, while both arms are extended horizontally. Gripping the ends of the elastic band with palms facing upward, the individual ensures that the upper arms remain closely aligned with the trunk. The exercise involves bending the elbows smoothly, maintaining the flexed position for a duration of 3-5 seconds, and subsequently relaxing back to the initial position. (b) Chest press in a sitting position employing a yellow elastic band in ICU: Assume a seated position, allowing the elastic band to wrap around below the scapulae and emerge from the armpits. Grasp the elastic band with both hands, choosing an appropriate width. Ensure the upper arms are firmly against the trunk, with the elbow joints flexed and the forearms in a neutral position, palms facing each other. Extend both arms forward, stretching the elastic band, and maintain this position for 3-5 seconds before relaxing and returning to the initial position. (c) Rowing in sitting position employing a red elastic band in the general ward. Assume a seated position, with the elastic band securely fixed in front of the body at approximately waist level. Adjust the sitting posture, ensuring the shoulders are relaxed and the arms are extended forward. Grasp both ends of the elastic band with palms facing each other, selecting an appropriate width. Maintain stability in the body, keeping the back straight, upper arms close to the trunk, and shoulders retracted while performing a chest-up movement. Maintain this position for 3-5 seconds before relaxing and returning to the initial position. (d) Chest press in standing position employing a red elastic band in the general ward: Assume a standing position and pass the elastic band behind the back. Grasp the elastic band with both hands, selecting an appropriate width. Ensure the upper arms are firmly against the trunk, while the forearms maintain a neutral position with palms facing each other. Extend both arms forward, reaching shoulder-level height. Once achieving the designated position, maintain it for 3-5 seconds before relaxing and returning to the initial position.

Table 1 Resistance training intervention profile

| **Indicators** | **Resistance Training group (N=28)** | **Combination group (N=28)** |
| --- | --- | --- |
| **Number of target completion sessions**[session, M(Q_25_,Q_75_)] | 8.0(5.75,12.25) | 10.0(8.0,14.0) |
| **Number of actual completion sessions** [session, M(Q_25_,Q_75_)] | 8.0(5.0,12.25) | 9.5(8.0,13.25) |
| **Compliance rate** [%, M(Q_25_,Q_75_)] | 90.60(75.0,100.0) | 100.0(83.46,100.0) |
| **Compliance rate≥80%**[N(%)] | 20(76.92) | 21(80.8) |
| **Cumulative session of unfinished interventions,** [N(%)] | 28(10.98) | 46(14.56) |
| weakness complaints [N(%)] | 10(35.71) | 15(32.60) |
| discomforts such as abdominal bloating and pain [N(%)] | 4(14.29) | 3(6.5) |
| dropouts [N(%)] | 8(28.57) | 13(28.26) |
| affected by the epidemic [N(%)] | 3(10.71) | 9(19.57) |
| other[N(%)] | 3(10.71) | 6(13.04) |
| **Training in the ICU** [day, M(Q_25_,Q_75_)] | 2.0(1.0,3.25) | 2.0(1.0,5.0) |
| **Training in the general ward** [day, M(Q_25_,Q_75_)] | 5.0(3.75,7.50) | 8.0(0.75,9.25) |
| **Duration per intervention** [min, M(Q_25_,Q_75_)] | 23.27(16.24,28.50) | 20.0(15.91,25.13) |
| **Number of training sessions in the supine position** [session, M(Q_25_,Q_75_)] | 3.0(1.0,4.25) | 4.5(2.75,6.75) |
| **Number of training sessions in the sitting position** [session, M(Q_25_,Q_75_)] | 3.0(2.0,4.25) | 3.5(0,5.0) |
| **Number of training sessions in the standing position** [session, M(Q_25_,Q_75_)] | 1.5(0,3.0) | 0(0,3.0) |
| **Proportion of training in the supine position**[%**,**M(Q_25_,Q_75_)] | 35.42(13.49,58.48) | 47.22(32.81,89.29) |
| **Proportion of training in the sitting position**[%**,**M(Q_25_,Q_75_)] | 37.5(22.31,55.95) | 23.37(0,56.25) |
| **Proportion of training in the standing position**[%**,**M(Q_25_,Q_75_)] | 25.0(0,35.0) | 0(0,18.40) |
| **Number of patient using resistance bands**[N(%)] | 20(76.92) | 22(84.62) |
| **Number of patient using yellow resistance bands** [N(%)] | 18(69.23) | 18(69.23) |
| **Number of patient using red resistance bands** [N(%)] | 13(46.42) | 13(46.42) |
| **Proportion of using elastic bands in ICU** [%**,**M(Q_25_,Q_75_)] | 0(0,62.6) | 54.5(0,100) |
| **Proportion of using elastic bands in general ward** [%**,**M(Q_25_,Q_75_)] | 77.5(17.5,100) | 76.4(50,100) |
| **Proportion of training using resistance bands** [%**,**M(Q_25_,Q_75_)] | 66.67(25.0,88.19) | 76.39(29.22,87.85) |
| **Proportion of training using yellow resistance bands** [%**,**M(Q_25_,Q_75_)] | 25.0(0,44.65) | 40.0(0,81.88) |
| **Proportion of training using red resistance bands** [%**,**M(Q_25_,Q_75_)] | 0(0,49.58) | 0(0,35.18) |
| **Adverse reactions related to exercise training** [N(%)] | 0(0) | 0(0) |

Note: for example, the proportion of training in the supine position was determined by calculating the ratio of training sessions in the supine position to the total training sessions for each individual, resulting in a continuously variable range of 0% to 100%. To assess the concentration and dispersion of this proportion across the entire group, we analyzed the median and IQR (Q_25_, Q_75_). Similarly, other metrics can be explained using the same approach.Table 2 HMB intervention profile.

| **Indicators** | **HMB group (N=28)** | **Combination group (N=28)** |
| --- | --- | --- |
| **Number of target completion days** [day, M(Q_25_,Q_75_)] | 12.0(8.0,19.50) | 10.5(7.0,16.75) |
| **Number of actual completion days** [day, M(Q_25_,Q_75_)] | 11.0(7.50,18.50) | 8.5(6.0,12.0) |
| **Number of target completion sessions** [session, M(Q_25_,Q_75_)] | 24.0(16.0,39.0) | 21.0(14.0,33.5) |
| **Number of actual completion sessions** [session, M(Q_25_,Q_75_)] | 22.0(14.5,37.5) | 18.0(13.0,25.5) |
| **Compliance rate** [%, M(Q_25_,Q_75_)] | 92.11(88.71,100.0) | 91.03(85.88,92.86) |
| **Compliance rate≥80%**[N(%)] | 27(96.43) | 26(92.86) |
| **Cumulative session of unfinished interventions,** [N(%)] | 70(9.3) | 93(13.2) |
| suspension due to gastrointestinal intolerance | 15(21.43) | 14(15.05) |
| miss taking | 25(35.71) | 32(34.40) |
| withdraw | 17(24.29) | 26(27.96) |
| other | 13(18.57) | 21(22.58) |
| **Analysis of the causes of study protocol deviations**[N(%)] | 70(9.3) | 93(13.2) |
| certainly related to HMB | 0 | 0 |
| possibly related to HMB | 10(14.29) | 8(8.60) |
| possibly unrelated to HMB | 8(11.43) | 20(21.51) |
| certainly unrelated to HMB | 36(51.43) | 50(53.76) |
| unable to judge | 16(22.86) | 15(16.13) |

Table 3 The results of GLLM analysis for comparison of body composition among four groups

| **Outcomes** | **T_0_**  $\boldsymbol{(}\bar{\boldsymbol{x}}\boldsymbol{\pm s)}$ | **T_1w_** | | **T_2w_** | | **Hospital discharge** | |
| --- | --- | --- | --- | --- | --- | --- | --- |
|  |  | $\bar{\boldsymbol{x}}\boldsymbol{\pm}\boldsymbol{s}$ | ***β(95%CI)*** | $\bar{\boldsymbol{x}}\boldsymbol{\pm}\boldsymbol{s}$ | ***β(95%CI)*** | $\bar{\boldsymbol{x}}\boldsymbol{\pm}\boldsymbol{s}$ | ***β(95%CI)*** |
| **FFM^a^** |  |  |  |  |  |  |  |
| RT group | 52.35±8.60 | 51.25±10.17 | 3.43(-1.765-8.625) | 49.53±10.2 | 0.918(-6.629-8.465) | 51.33±9.98 | 4.053(-1.042-9.149) |
| HMB group | 51.19±9.65 | 50.18±12.88 | 2.39(-2.853-7.634) | 50.98±7.14 | 2.273(-4.192-8.738) | 48.81±12.49 | 1.565(-3.578-6.708) |
| Combination group | 50.83±8.09 | 49.22±7.98 | 1.373(-3.823-6.569) | 50.35±6.60 | 1.162(-6.788-9.113) | 50.19±7.82 | 2.882(-2.214-7.978) |
| Control group | 48.52±8.06 | 47.84±8.24 | reference | 48.50±8.33 | reference | 47.30±8.27 | reference |
| *F* | 1.012 | 0.613 |  | 0.161 |  | 0.905 |  |
| *P* | 0.387 | 0.607 |  | 0.923 |  | 0.439 |  |
| **ASMM^b^** |  |  |  |  |  |  |  |
| RT group | 20.29±4.03 | 19.93±4.89 | 1.415(-0.982-3.811) | 19.46±4.40 | -0.226(-5.016-4.563) | 20.31±4.96 | 1.538(-0.933-4.009) |
| HMB group | 19.98±5.01 | 19.70±5.12 | 1.186(-1.233-3.605) | 20.30±5.43 | 0.617(-3.494-4.728) | 18.57±4.52 | -0.195(-2.689-2.299) |
| Combination group | 20.45±6.81 | 18.7±3.54 | 0.188(-2.209-2.584) | 19.70±3.67 | 0.10(-4.935-5.135) | 19.36±3.48 | 0.593(-1.878-3.064) |
| Control group | 18.84±4.09 | 18.51±4.70 | reference | 20.5±5.70 | reference | 18.77±5.69 | reference |
| *F* | 0.578 | 0.665 |  | 0.049 |  | 0.751 |  |
| *P* | 0.630 | 0.574 |  | 0.986 |  | 0.522 |  |
| **SMI^c^** |  |  |  |  |  |  |  |
| RT group | 9.65±1.69 | 9.47±1.82 | 0.228(-0.798-1.254) | 9.49±1.95 | 0.698(-1.21-2.606) | 9.59±1.98 | 0.270(-0.766-1.306) |
| HMB group | 9.76±2.15 | 9.61±2.31 | 0.374(-0.661-1.410) | 10.03±2.30 | 1.176(-0.458-2.81) | 9.11±1.88 | -0.205(-1.251-0.841) |
| Combination group | 9.72±1.80 | 9.22±1.57 | -0.029(-1.055-0.998) | 10.0±1.83 | 1.275(-0.735-3.285) | 9.62±1.66 | 0.296(-0.741-1.332) |
| Control group | 9.60±1.99 | 9.28±2.11 | reference | 8.95±1.78 | reference | 9.36±2.37 | reference |
| *F* | 0.058 | 0.263 |  | 0.847 |  | 0.395 |  |
| *P* | 0.981 | 0.852 |  | 0.469 |  | 0.757 |  |
| **PhA^d^** |  |  |  |  |  |  |  |
| RT group | 5.24±1.05 | 5.57±1.14 | 0.609(0.002-1.217) | 5.1±1.18 | -1.534(-4.292-1.225) | 5.73±1.21 | 0.787(0.229-1.345)^**^ |
| HMB group | 4.7±0.91 | 4.92±0.94 | -0.049(-0.662-0.564) | 4.76±0.95 | -1.929(-4.296-0.439) | 4.98±0.78 | 0.027(-0.536-0.591) |
| Combination group | 4.55±0.86 | 4.88±1.19 | -0.07(-0.678-0.537) | 4.95±1.02 | -1.578(-4.48-1.323) | 5.12±1.0 | 0.20(-0.370-0.770) |
| Control group | 4.88±1.25 | 4.98±1.37 | reference | 5.15±4.93 | reference | 4.96±1.22 | reference |
| *F* | 2.101 | 2.188 |  | 0.968 |  | 3.288 |  |
| *P* | 0.10 | 0.089 |  | 0.408 |  | 0.021 |  |

GLLM results of the main effect and interaction effect：

a FFM model: Fat Free Mass, group: *F*=1.675*，P*=0.172, time: *F*=0.474，*P*=0.7, group × time interaction: *F*=0.123*，P*=0.999；

b ASMM model: Appendicular Skeletal Muscle Mass, group: *F*=0.584, *P*=0.626, time: *F*=0.504*, P*=0.68, group × time interaction: *F*=0.281*, P*=0.98；

c SMI model: skeletal muscle index, group: *F*=0.737, *P*=0.530, time: *F*=0.552*，P*=0.647, group × time interaction: *F*=0.393*, P*=0.938；

d PhA: phase angle, group:*F*=1.364, *P*=0.254, time: *F*=2.390*, P*=0.069, group × time interaction: *F*=0.576*, P*=0.816；

***P<0.05, **** P<0.01,***** P<0.001

Table 4 The results of GLLM analysis for comparison of psychological and cognitive function among four groups

| **Outcomes** | **T0**  $\boldsymbol{(}\bar{\boldsymbol{x}}\boldsymbol{\pm s)}$ | **ICU discharge**  $\boldsymbol{(}\bar{\boldsymbol{x}}\boldsymbol{\pm s)}$ | **Hospital discharge**  $\boldsymbol{(}\bar{\boldsymbol{x}}\boldsymbol{\pm s)}$ | **1 month follow up**  $\boldsymbol{(}\bar{\boldsymbol{x}}\boldsymbol{\pm s)}$ |
| --- | --- | --- | --- | --- |
| **HADS-A^a^** |  |  |  |  |
| RT group | 9.50±3.77 | 7.21±3.64 | 4.79±3.44 | 4.36±2.75 |
| HMB group | 7.57±4.85 | 7.18±3.98 | 5.0±3.88 | 3.92±3.08 |
| Combination group | 7.0±5.13 | 6.39±4.98 | 4.57±4.31 | 4.38±4.06 |
| Control group | 9.46±5.3 | 6.69±4.83 | 4.88±3.42 | 4.84±3.16 |
| *F* | 2.623 | 0.344 | 0.174 | 0.352 |
| *P* | 0.05 | 0.794 | 0.914 | 0.788 |
| **HADS-D^b^** |  |  |  |  |
| RT group | 10.0±4.20 | 7.14±4.37 | 5.07±3.90 | 4.14±2.81 |
| HMB group | 9.43±4.41 | 8.57±5.13 | 6.57±5.01 | 4.88±2.83 |
| Combination group | 8.7±5.37 | 6.93±4.91 | 4.96±4.17 | 4.88±4.46 |
| Control group | 10.18±4.97 | 7.73±5.29 | 5.81±4.23 | 4.76±3.21 |
| *F* | 0.559 | 0.628 | 0.819 | 0.306 |
| *P* | 0.642 | 0.597 | 0.484 | 0.821 |
| **MMSE^c^** |  |  |  |  |
| RT group | 23.7±6.256 | 25.57±6.13 | 27.57±4.44 |  |
| HMB group | 21.6±5.58 | 24.15±4.91 | 26.18±3.78 |  |
| Combination group | 21.62±7.47 | 22.46±7.73 | 24.54±5.94 |  |
| Control group | 20.74±6.41 | 23.16±5.77 | 24.73±5.87 |  |
| *F* | 1.161 | 1.354 | 2.306 | - |
| *P* | 0.325 | 0.257 | 0.077 | - |

a HADS-A: Anxiety subscale of Hospital Anxiety and Depression Scale, group: *F*=1.711, *P*=0.164, time: *F*=24.718, *P*<0.001, group × time interaction: *F*=0.672, *P*=0.734；

b HADS-D: Depression subscale of Hospital Anxiety and Depression Scale, group: *F*=1.220, *P*=0.302, time: *F*=29.139, *P*<0.001, group × time interaction: *F*=0.402, *P*=0.934；

c MMSE: Mini-Mental State Examination, group: *F*=4.158, *P*=0.007, time: *F*=12.201, *P*<0.001, group × time interaction: *F*=0.131, *P*=0.992；

**P*<0.05, ***P*<0.01, ****P*<0.001
